# Overwork: "A Sad Device of the Devil"

**Published:** 1888-12-15

**Authors:** 


### The Hospital

A Weekly Institutional Journal of

Science, Medicine, Nursing, and Philanthropy.

*For Hospitals, Asylums, Medical Practitioners, Students, Nurses, Families, Parishes, Congregations, and the Charitable Public.*

Vol. V. No. 116.] [December 15, 1888.

### Overwork: "A Sad Device of the Devil."

"Criticus Parvulus" will probably demand that we first prove the existence of the Devil, and then take into consideration the question of his devices. For our present purpose it does not matter whether he exists or not. It may, however, be not inappropriate to remind "Criticus Parvulus" that some people almost as learned, as able, and as weighty in judgment as himself, have honestly believed in the Devil. To say nothing of St. Paul and St. Augustine, there are names to be mentioned which the world has agreed to hold in some respect for learning and genius. Dante was not a fool, nor was he without a varied experience; Milton is known to have lived on a high level of intellectual earnestness and moral practice; Newton, if Macaulay's judgment goes for anything, was a miracle of genius and worth; Macaulay himself was no schoolboy; the last President of the British Association must have been at least an eminent man of science, or the men of scientific light and leading would have had none of him. All those persons believed in the Devil. "Criticus Parvulus" should remember that.

The controversial aspect of the title of this article may be passed by. The subject matter has to do with opinions expressed in the year 1829. There may have been a Devil then; but perhaps modern science, as it has accomplished so many great deeds, has succeeded in capturing and killing him since that date. If so, we, as well as others, will rejoice. There can be no special reason why any of us should wish his life to be unduly prolonged. In the year 1829, on the 27th of December of that year, the Rev. Charles Simeon, of Cambridge, wrote a letter to a friend. That letter has been preserved; and from a physiological standpoint it was well worth preserving, since it anticipates a good deal of the practical physiology of the present day. If it be asked how a clergyman should know anything about physiology, the answer is, that every clergyman and every other man has a thoroughly equipped physiological laboratory in his own person. Does he not eat; does he not digest; does he not sleep; does he not walk or run; does he not live and die? What physiological process which belongs to man is foreign to him? And has he not got eyes and ears, and a nose, and fingers, and a brain? and can he not employ these with understanding in the careful, accurate, and intelligent observation of himself and his own doings? For hundreds of years the clergy thought theology belonged to the study, and not to the market-place. Now the doctor is wallowing in the same stupid heresy, and thinks physiology belongs to the laboratory and not to the individual man. Alas for the progress of the species! Does it not look as if the movement were in somewhat of a circular direction?

Simeon believed in the Devil thoroughly, and believed moreover that he was an exceedingly subtle personage. He did not hold that man was mere "organisation in action," like a thrashing machine at work, or a mousetrap at the moment when the cheese is nibbled. A man to him was a soul, an immortal; and this life was the scene of a tragic play. For forty, fifty, or four-score years the subtle Devil played against the man, and the stake was an immortal soul. Even Goethe recognised that for purposes of art, both the Devil and the immortal soul might have their uses. Simeon writes to a brother clergyman that he hears of one of his clerical friends who is "destroying his health by over-work." "Truly," says he, "this is a sad device of the Devil." Now whether all can agree on that point or not, there can be no controversy about this—that to destroy the health is a bad thing in itself. Granted that in some rare instances it is, like bankruptcy, unavoidable, yet those instances must be very rare indeed. A man who destroys his health by over-work, in nineteen cases out of twenty does a foolish thing; and to the extent that the thing is foolish, to that extent it is not virtuous; to that extent wise men will disapprove; and, as we are writing about clergymen, we may be permitted to remind them and all religious persons, that just as reasonable men cannot approve of broken-down health, which is the result of want of good sense and judgment, so it may be presumed that Almighty God himself cannot and will not approve. There is, as a rule, no merit in it, but a very great deal of demerit.

Clergymen are sometimes very unwilling to listen to doctors. Perhaps, however, they will be more respectful to a portion of the experience of a clerical brother and father. Simeon, when he wrote our letter, was nearly seventy years of age. He gives in it a brief outline of his physiological life. "I was guarded in my youth," he says, "by a pious friend, and by attending to his admonitions, I went on for twenty-four years without ever being laid by for a single Sunday; and had I been aware that I was breaking down at the end of that time, I would have at once reduced my labours within my strength. But never having broken down yet, I thought I might disregard the symptoms which I had hitherto noticed; and when my chest was sore, or my voice hoarse, or my body feverish, I smiled at it, and the Devil smiled at me. Yes! he had me at last, and for thirteen years I was laid aside." Thirteen years disabled ! If Simeon had been a man in business, or the minister of an unendowed Church, or a curate, those thirteen years would have been as effectual as thirteen hundred in stopping his career. He and his family would probably have had thirteen years of starvation, with no possibility of ever regaining position or influence. Probably no man breaks down with a light heart. His policy is not to break down at all.

To stop work means for most men a check in their onward progress. They cannot contemplate such a check with anything but pain and dismay. They have marked out the lines of their career, and, like the driver of an express train, they hate to shut off steam. But supposing the express to have got on to a wrong line in the darkness, whilst nobody knows anything about it; and supposing another express to be approaching from an opposite direction twenty miles away, will a sane driver refuse to shut off steam, and madly push on to the tremendous catastrophe? The illustration is not inapt. Many a man in the difficult conduct of life gets himself on to wrong lines—lines of overwork, of worry, of stress—which it is impossible for him to sustain. Whether is better: to stop now, and brave the check and the present loss, or to wilfully persist until broken health, or lunacy, or death, prove to be the final alternative? The annals of modern medicine record thousands of tragedies, which only await their Shakespeare or their Göethe to make them live for ever in the saddest literature of the world.

Men think they cannot stop at the present moment because duty forbids. In a few cases no doubt it is so; and individuals have to die as well as to live for their families. But before a man makes up his mind to that, he should be very sure that it is his duty. As Simeon says: "The Devil gets on his side—first our conscience; secondly our self-approbation and self-complacency; and with these two heifers he plows till he has entirely prevailed agsinst us." This may be considered a mere quaint conceit by some, and unworthy of serious attention. Have they not brains enough to perceive that the thing is as true in its essence as if it were stated with the strictest mathematical precision, or with all the scented pocket-handkerchief nicety of a poem in a society magazine? Many a man's uninstructed conscience and too great self-approbation prove his physiological ruin. In this paper our constant plea is for "sense." There is nothing the present age needs more. Conscience is not wanting; knowledge is not wanting; but sense seems to have broken both his legs and to limp far in the rear on the most antediluvian of crutches. As Tennyson long ago said, "Knowledge comes, but wisdom lingers." This age knows all about everything—about physiology and the laws of health as well as other things. But in actual practice it is not more rational, if indeed it be so rational, as the contemporaries of Shakespeare and the Tudors.

One or two of Simeon's homely illustrations may properly conclude this article. "Conscience argues thus," he says:—"I can do more, and therefore I ought to do more. Now go and argue this at a well-covered table—'I can eat more, yes, and I can eat still more,' and you will find at last the result of it." Or, "you have got two sacks of corn to carry a mile within the week; will you go and put them both on your back at once, or carry them both in one day?" If a man have a four-mile heat to run, will he "run the first mile as if that were to close the race?" He will say, "No, I have four miles to run, and I must husband my strength for the fourth mile." It was all as plain as the nose upon one's face to Simeon, sixty years ago. It is as plain to us now—to clergymen and lawyers and doctors and men of business and everybody else. We are not so stupid as not to know it. What we want is the clear judgment and the firm will to say—"Come what will, my life must be spent so as to secure its proper proportion of rest, recreation, and sleep, as well as its period of work." Every man should so live as that at sixty or seventy he may be able to give up his strenuous labours with a constitution unimpaired, and with such a degree of health as will enable him thoroughly to enjoy a "green and pleasant old age."

